# Electrophysiological basis of cardiac arrhythmia in a mouse model of myotonic dystrophy type 1

**DOI:** 10.3389/fphys.2023.1257682

**Published:** 2023-09-21

**Authors:** Vamsi Krishna Murthy Ginjupalli, Michael Cupelli, Jean-Baptiste Reisqs, Yvonne Sleiman, Nabil El-Sherif, Genevieve Gourdon, Jack Puymirat, Mohamed Chahine, Mohamed Boutjdir

**Affiliations:** ^1^ Cardiovascular Research Program, VA New York Harbor Health care System, Brooklyn, NY, United States; ^2^ Departments of Medicine, Cell Biology and Pharmacology, SUNY Downstate Health Sciences University, Brooklyn, NY, United States; ^3^ Department of Medicine, Faculty of Medicine, Université Laval, Quebec City, QC, Canada; ^4^ Centre de recherche en Myologie, Inserm, Institut de Myologie, Sorbonne Université, Paris, France; ^5^ LOEX, CHU de Québec-Université Laval Research Center, Quebec City, QC, Canada; ^6^ CERVO Research Center, Institut Universitaire en Santé Mentale de Québec, Quebec City, QC, Canada; ^7^ Department of Medicine, New York University, Grossman School of Medicine, New York, NY, United States

**Keywords:** myotonic dystrophy type 1, electrophysiology, ECG, arrhythmia, ion channels, conduction defects

## Abstract

**Introduction:** Myotonic dystrophy type 1 (DM1) is a multisystemic genetic disorder caused by the increased number of CTG repeats in 3′ UTR of *Dystrophia Myotonia Protein Kinase (DMPK)* gene. DM1 patients experience conduction abnormalities as well as atrial and ventricular arrhythmias with increased susceptibility to sudden cardiac death. The ionic basis of these electrical abnormalities is poorly understood.

**Methods:** We evaluated the surface electrocardiogram (ECG) and key ion currents underlying the action potential (AP) in a mouse model of DM1, DMSXL, which express over 1000 CTG repeats. Sodium current (I_Na_), L-type calcium current (I_CaL_), transient outward potassium current (I_to_), and APs were recorded using the patch-clamp technique.

**Results:** Arrhythmic events on the ECG including sinus bradycardia, conduction defects, and premature ventricular and atrial arrhythmias were observed in DMSXL homozygous mice but not in WT mice. PR interval shortening was observed in homozygous mice while ECG parameters such as QRS duration, and QTc did not change. Further, flecainide prolonged PR, QRS, and QTc visually in DMSXL homozygous mice. At the single ventricular myocyte level, we observed a reduced current density for I_to_ and I_CaL_ with a positive shift in steady state activation of L-type calcium channels carrying I_CaL_ in DMSXL homozygous mice compared with WT mice. I_Na_ densities and action potential duration did not change between DMSXL and WT mice.

**Conclusion:** The reduced current densities of I_to_, and I_CaL_ and alterations in gating properties in L-type calcium channels may contribute to the ECG abnormalities in the DMSXL mouse model of DM1. These findings open new avenues for novel targeted therapeutics.

## Graphical Abstract

**Figure:**
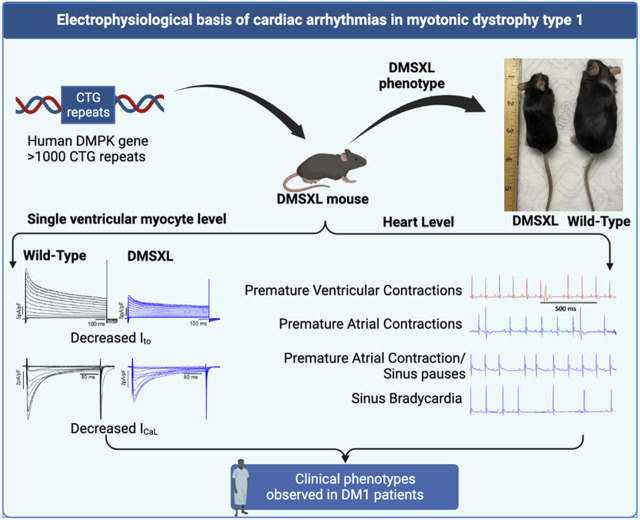

## Introduction

Myotonic dystrophy or dystrophia myotonica type 1 (DM1), also known as Steinert disease, is a multisystemic disorder that affects skeletal and smooth muscles as well as the eye, central nervous system, endocrine system, and the cardiac muscle (Steinert, 1909; Ranum and Day, 2004). DM1 is the most common adult form of muscular dystrophy and is caused by the expansion of cytosine-thymine-guanine (CTG) repeats in the trinucleotide of the untranslated region (3′ UTR) of the *Dystrophia Myotonia Protein Kinase (DMPK)* gene located on chromosome 19q13.3 (Mahadevan et al., 1992; Bird, 1993). Healthy individuals typically have CTG repeats ranging from 5 to 27, while in the pathogenic conditions the *DMPK*-expanded alleles contain more than 50 CTG repeats (Brook et al., 1992; Gennarelli et al., 1996). The size of the CTG repeat, which tends to increase with age and can sometimes result in very large expansions, is generally correlated with the severity of the disorder and the age at which symptoms first appear (Harper et al., 1992; Hunter et al., 1992). This correlation provides a molecular explanation for the phenomenon of anticipation, which is commonly observed in families affected by DM1 (Harper et al., 1992). Studies suggest that the majority of symptoms in DM1 are a result of a toxic RNA gain-of-function caused by the accumulation of mutant DMPK RNA in ribonuclear foci in the nucleus, resulting in the sequestration of MBNL1 (Miller et al., 2000; Mahadevan et al., 2021), which is brought on by the expanded CUG repeat (Lee and Cooper, 2009; Chau and Kalsotra, 2015).

The electrical abnormalities on the surface electrocardiogram (ECG) are detected in 80% of DM1 patients and are the second most common cause of DM1 deaths after respiratory complications (D'Andrea et al., 2009; Mankodi and Thornton, 2002). The primary clinical cardiac manifestation in DM1 is the development of conduction disturbances with progressive atrioventricular (AV) block and ventricular arrhythmias, including tachycardia, bradycardia, and sudden cardiac death (SCD) (Petri et al., 2014). While extensive progress has been made in DM1 skeletal muscle pathophysiology, the cardiac arrhythmogenic aspects of DM1 remain elusive despite the high prevalence of cardiac arrhythmias and SCD in DM1 patients (Petri et al., 2014). Currently, no pharmacological treatment is available for these patients. The implantation of a pacemaker or defibrillator to prevent sudden death remains the only available treatment for DM1 patients (Laurent et al., 2011).

The DMSXL model of DM1 is a transgenic mouse model that constitutively expresses the human DM1 locus under the regulation of its own promoter and its *cis* regulatory elements (Huguet et al., 2012). The originality of this model lies in the pattern of expression of the human *DMPK* gene in DMSXL which is similar to DM1 patients including a higher level of expression in the heart compared to other tissues. This model displays clinical features similar to those observed in the human disease including reduced muscle strength, lower motor performances, peripheral neuropathy, and respiratory impairments (Seznec et al., 2000; Huguet et al., 2012; Panaite et al., 2013). We leveraged this DMSXL model of DM1 to thoroughly examine the role of key ion channel changes in the arrhythmogenesis of DM1 both at the surface ECG level and in ventricular myocytes as well.

## Materials and methods

All research involving mice was conducted in accordance with the procedures and guidelines set forth by The Guide for the Care and Use of Laboratory Animals (National Research Council Committee for the Update of the Guide for the Care and Use of Laboratory Animals, 2011 revised 2011) (Animals and Use of, 2011). The experimental protocols were approved by the Institutional Animal Care and Use Committee of the Veterans Affairs New York Harbor Healthcare System (New York, NY). The study was also carried out in compliance with the ARRIVE guidelines 2010 (Kilkenny and Altman, 2010).

### DMSXL colony

The DM1 patient’s 45 kilobase genomic fragment containing the *DMPK* gene was employed to create a homozygous DMSXL transgenic mouse model as previously described (Seznec et al., 2000). This model features an expansion of over 1000 CTG repeats (Huguet et al., 2012). The DMSXL colony was divided into three genetic classifications: homozygous (Homo), heterozygous (Het), and the reference wild type (WT). Genotyping was performed via qPCR locally and verified by a 3rd party automated genotyping service (TransnetYX, Cordova, TN) (Supplementary Table S1). Because worse electrical outcomes are seen in older patients (Mathieu et al., 1999) we used 12- to 20-month-old DMSXL mice (male and female).

### Electrocardiogram

High resolution surface ECG was recorded and analyzed using a digital acquisition and analysis system (AD Instruments, Colorado Springs, CO). Mice were placed on a warm pad and continuous anesthetic agent isoflurane (2%–3%) was used with a SomnoSuite Low-Flow Anesthesia System (Kent Scientific, Torrington, CT). Needle electrodes were inserted sub-dermally at the wrist and ankle. Electrical signals were recorded at 2000 Hz, stored in a computer hard drive, and analyzed off-line using LabChart 8.0 software (AD Instruments). Tracings were analyzed for heart rate, PR interval, QT interval, QRS duration, conduction abnormalities including first-, second-, and third-degree AV block, and arrhythmic events when applicable (Ripplinger et al., 2022). The heart rate corrected QT interval (QTc) was calculated using the modified Bazett’s formula (Mitchell et al., 1998). In a subset of mice, baseline ECG recordings were taken for a period of 2 min, then flecainide acetate was administered via intraperitoneal injection at a dosage of 20 mg/kg body weight (Algalarrondo et al., 2015; Tylock et al., 2020), and ECG recordings continued for 20 min post-administration.

### Ventricular myocyte isolation

Ventricular cardiomyocytes were isolated from the hearts of DMSXL and WT mice after excision and cannulation via the aorta using the Langendorff technique (Mancarella et al., 2008; Karnabi et al., 2011). The heart was perfused with nominally Ca^2+^ free Tyrode solution containing (in mM): 130 NaCl, 5 KCl, 0.5 NaH_2_PO_4_, 10 HEPES, 10 Glucose, 10 2,3-Butanedione monoxime, 10 Taurine, and 1 MgCl (pH 7.4). After the blood was washed out, hearts were then perfused with digestion buffer containing collagenase II (0.5 mg/mL), collagenase IV (0.5 mg/mL) (Worthington, United States), and protease type XIV (0.2 mg/ml; Sigma, St. Louis, MO, United States). When the heart was digested, the atria were removed, and ventricles gently minced with forceps to dissociate single cardiomyocytes in stop buffer containing 5% FBS in perfusion buffer. Ca^2+^ was gradually introduced and increased from 0.1 mM to 1 mM. Isolated cardiomyocytes were maintained in M199 medium and subsequently used for patch-clamp recordings.

### Patch clamp

Whole cell configuration of the patch-clamp technique was used for ion current recordings. A minimum of 4 mouse hearts were used for each group, and the reported *n* corresponds to the number of cells for each group. Cells were first superfused with Tyrode’s solution and then switched to an appropriate solution for each current studied as previously described (Mancarella et al., 2008). All experiments were conducted at room temperature. For the transient outward potassium current (I_to_) recordings, the external solution contained (in mM): 140 CoCl_2_, 5.4 KCl, 1.8 CaCl_2_, 1 MgCl_2_, 10 HEPES, 5 Glucose, and 3 CoCl_2_ (pH 7.4 with NaOH). The pipette solution for I_to_ contained (in mM): 130 KCl, 12 NaCl, 1 MgCl_2_, 10 EGTA, 4 Mg-ATP, and 1 CaCl_2_ (pH 7.2 with KOH). I_to_ were evoked by inactivating sodium channels using a 5 ms pre-pulse (Boutjdir et al., 1998; Rougier et al., 2019).

Pipette solution for the L-type calcium current, I_CaL_ recordings contained (in mM): 139.8 CsCl, 10 K-EGTA, 4 MgCl_2_, 0.062 CaCl_2_, 5 Na^2^-creatine phosphate, 10 HEPES, 3.1 Mg ATP, and 0.42 Mg GTP (pH 7.4 with CsOH). The external solution for I_CaL_ recordings contained (in mM): 132 NaCl, 5.4 CsCl, 1.8 CaCl_2_, 1.8 MgCl_2_, 0.6 NaH2PO4, 5 4-amino-pyridine, 10 HEPES, 5 dextrose, and 5 Na-pyruvate (pH 7.4 with NaOH). The sodium current (INa) and T-type Ca^+^ currents (I_CaT_) were blocked by a pre-pulse to −40 mV from a holding potential of −90 mV every 10 s (Mancarella et al., 2008).

Pipette solution for the sodium current (I_Na_) recordings contained (in mM): 70 Cs-Aspartate, 20 CsCl, 1 MgCl_2_, 11 EGTA, 5 Mg-ATP, and 1 CaCl_2_ (pH 7.2 with CsOH). The external solution for I_Na_ recordings contained (in mM): 5 NaCl, 125 NMDG, 5 CsCl, 1.2 MgCl_2_, 2 CaCl_2_, 10 HEPES, and 10 Dextrose (pH 7.4 with CsOH). Nifedipine and CoCl_2_ and were added to the external solution to block I_CaL_ and the I_CaT_, respectively. I_Na_ was evoked with 30 ms duration pulses to +30 mV from a holding potential of −120 mV at 5 s intervals.

For the action potential (AP) recordings, cardiomyocytes were bathed in a solution containing (in mM) 140 NaCl, 5.4 KCl, 1.8 CaCl_2_, 1.2 MgCl_2_, 10 HEPES, and 5 glucose (pH 7.4 with NaOH). Cardiomyocytes were initially voltage-clamped (holding potential −80 mV) and the patch electrode contained (in mM) 120 KCl, 1.5 CaCl_2_, 5.5 MgCl_2_, 5 Na-ATP, 5 K-EGTA, and 10 HEPES (pH 7.2 with KOH). APs were then evoked at 1 Hz with rectangular pulses in current-clamp mode. AP recordings were digitized at a sampling frequency of 10 kHz.

### Western blotting

Proteins from ventricular of WT and DMSXL mice were extracted as previously described (Srivastava et al., 2020). Briefly, ventricular tissue from WT and DMSXL mice were minced with pestles and homogenized using 500 µl RIPA Buffer (ThermoScientific #89900, Walthman, MA, United States) and a protease inhibitor cocktail (Sigma #P2714-1BTL, St. Louis, MO, United States) for 5 min on ice. Cell lysate was then incubated for 30 min at 4°C. The extracted samples were then centrifuged for 15 min at 14,000 g at 4°C. The samples were denatured in sample buffer for 30 min at 37°C, then resolved on 7.5% SDS-PAGE standard gel, and transferred onto a nitrocellulose membrane (BioRad Laboratories #1620145, Hercules, CA, United States). Blot was then blocked with Intercept-TBS Blocking Buffer (LI-COR #927–60001, Lincoln, NE, United States) for 1 h at room temperature, then incubated with the following primary antibodies overnight at 4°C to separate the proteins: anti-Ca_v_1.2 (Sigma #C1241; 1/150, St. Louis, MO, United States), anti-K_v_4.2 (Milipore #AB5360; 1/150, St. Louis, MO, United States), and anti-GAPDH (Sigma #G9545; 1/2000, St. Louis, MO, United States). The Odyssey CLx Imaging system (LI-COR, Lincoln, NE, United States) was used to develop immunoblots with IR-labeled secondary antibodies (1:20,000 dilution) for 1 h at room temperature. After developing the secondary antibody, the band intensity was quantified using ImageStudio and normalized to the GAPDH.

## Statistical analysis

### Body weight data

Data are reported as and mean ± standard error. Multiple comparisons between WT, Het, and Homo groups were performed using ANOVA and Tukey’s *post hoc* test. *p < 0.05; **p < 0.01; ***p < 0.001; ****p < 0.0001 (WT vs. Homo) and #*p* < 0.05, ##*p* < 0.01, ###*p* < 0.001; ####*p* < 0.0001 (Het vs. Homo). The software Prism8 (GraphPad, San Diego, CA, United States) was utilized for conducting all statistical analyses.

### Electrocardiographic data

Data are reported as and mean ± standard error. Multiple comparisons of heart rate, PR interval, QRS interval, and QTc between WT, Het, and Homo groups were performed using ANOVA and Tukey’s *post hoc* test. *p < 0.05; **p < 0.01; ***p < 0.001; ****p < 0.0001 (WT vs. Homo) and #*p* < 0.05, ##*p* < 0.01, ###*p* < 0.001; ####*p* < 0.0001 (Het vs. Homo). The software Prism8 (GraphPad, San Diego, CA, United States) was utilized for conducting all statistical analyses.

### Patch clamp data

Data are reported as and mean ± standard error. I_to_, I_CaL_ and I_Na_ amplitude, and APs were analyzed offline using Clampfit 10.8. AP durations at 10%, 30%, 50%, and 90% repolarization were measured. *p < 0.05; **p < 0.01; ***p < 0.001; ****p < 0.0001 (WT vs. Homo) and #*p* < 0.05, ##*p* < 0.01, ###*p* < 0.001; ####*p* < 0.0001 (Het vs. Homo). Multiple comparisons between WT, Het, and Homo groups were performed using ANOVA and Tukey’s *post hoc* test. The software Prism8 (GraphPad, San Diego, CA, United States) was utilized for conducting all statistical analyses.

### Western blot data

Data are reported as and mean ± standard error. Multiple comparisons between WT, Het, and Homo groups were performed using ANOVA and Tukey’s *post hoc* test. *p < 0.05; **p < 0.01; ***p < 0.001; ****p < 0.0001 (WT vs. Homo) and #*p* < 0.05, ##*p* < 0.01, ###*p* < 0.001; ####*p* < 0.0001 (Het vs. Homo). The software Prism8 (GraphPad, San Diego, CA, United States) was utilized for conducting all statistical analyses.

## Results

### Growth retardation in DMSXL mice

**FIGURE 1:**
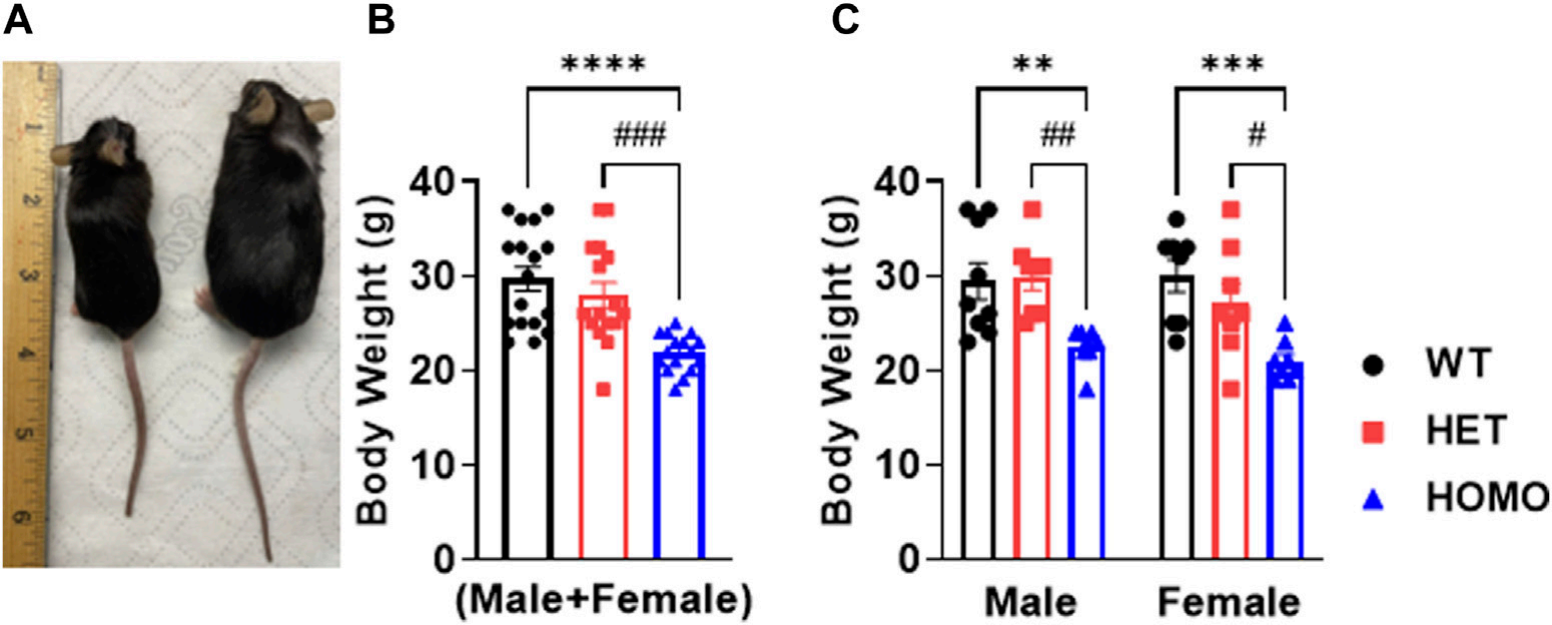
DMSXL mice showed growth retardation: Homozygous (Homo) mouse (left) and wild type (WT) mouse (right) at 13 months old **(A)**. Body weight of WT, heterozygous (Het), and Homo mice **(B)**. Comparative body weight distribution among male and female mice of both WT and DMSXL mice **(C)**. Bars indicate SEM. **p < 0.01; ***p < 0.001; ****p < 0.0001 (WT vs. Homo) and #*p* < 0.05, ##*p* < 0.01, ###*p* < 0.001 (Het vs. Homo) as determined by ANOVA and Tukey’s *post hoc* test.

Compared to WT and Het, Homo mice showed abnormal body growth as exemplified in lower body weight (WT 30.29 ± 1.1 g, n = 17; Het 27.72 ± 1 g, n = 16; Homo 21.88 ± 0.5 g, n = 16) (Figures 1A,B; Table 1), which was observed in both sexes. (Figure 1C; Table 2).

**TABLE 1:** Electrocardiographic measurements in wild type and DMSXL mice at baseline.

	Wild type (n = 17)	Heterozygous (n = 16)	Homozygous (n = 16)
Heart rate (BPM)	497.60 ± 10.2	516.31 ± 9.7	530.40 ± 9.1
PR Interval (ms)	45.32 ± 1.2	44.13 ± 1.2	40.28 ± 1.2*
QRS Interval (ms)	14.77 ± 0.5	14.69 ± 0.6	14.64 ± 0.5
QTc (ms)	45.39 ± 1.1	43.55 ± 0.9	41.92 ± 0.9
Body weight (g)	29.71 ± 1.3	28.00 ± 1.3	21.88 ± 0.5**** ###

BPM: Beats per minute. Values are means ± SE. *p < 0.05; ****p < 0.0001 (WT vs. Homo) and ###*p* < 0.001 (Het vs. Homo) as determined by ANOVA, and Tukey’s *post hoc* test.

**TABLE 2:** Sex-specific electrocardiographic measurements in wild type and DMSXL mice at baseline.

	Wild type (n = 17)		Heterozygous (n = 16)		Homozygous (n = 16)	
	Male (n = 9)	Female (n = 8)	Male (n = 8)	Female (n = 8)	Male (n = 8)	Female (n = 8)
Heart rate (BPM)	486.4 ± 15.61	498.8 ± 14.2	527.00 ± 12.1	505.50 ± 15.1	527.2 ± 13.7	529.3 ± 10.1
PR Interval (ms)	43.82 ± 1.4	46.95 ± 1.2	45.29 ± 1.8	42.97 ± 1.7	39.62 ± 2.2	40.94 ± 1.0*
QRS Interval (ms)	13.16 ± 0.8	16.20 ± 1.1	14.87 ± 1.0	14.51 ± 0.7	14.51 ± 0.6	14.77 ± 0.8
QTc (ms)	44.21 ± 1.3	46.67 ± 2.6	43.89 ± 1.7	43.2 ± 1.1	40.42 ± 0.8	43.42 ± 1.3
Body weight (g)	29.44 ± 1.9	30.0 ± 1.7	29.88 ± 1.4	27.13 ± 1.3	22.5 ± 0.5** #	21.00 ± 0.7*** ##

BPM: Beats per minute. Values are means ± SE. *p < 0.05; **p < 0.01; ***p < 0.001 (WT vs. Homo) and #*p* < 0.05; ##*p* < 0.01 (Het vs. Homo) as determined by ANOVA, and Tukey’s *post hoc* test.

### DMSXL homo mice showed spontaneous arrhythmic events and exhibited increased propensity for arrhythmic events with flecainide

**FIGURE 2:**
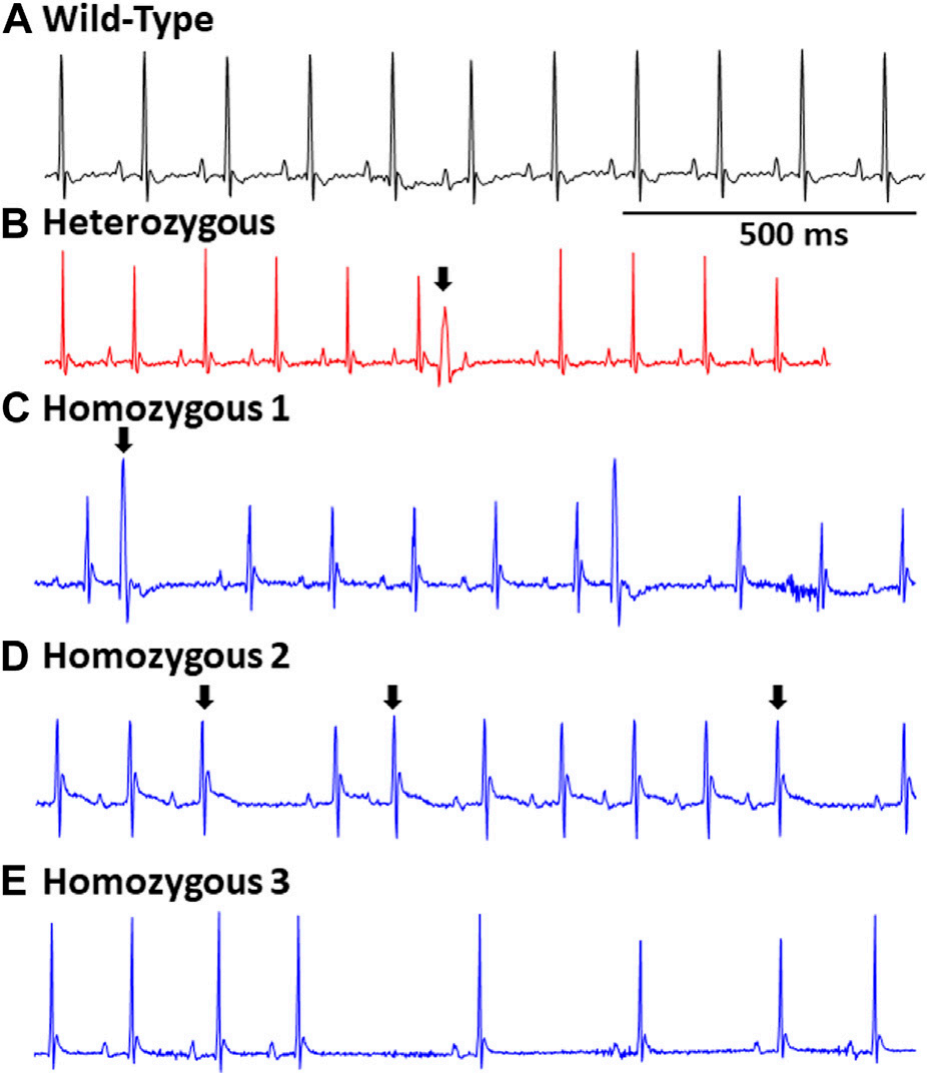
Representative EGCs in wild type and DMSXL mice: Standard ECG reading from a wild type mouse **(A)**. A heterozygous mouse ECG showing a premature ventricular contraction (PVC), (indicated by an arrow) **(B)**. A Homo mouse ECG demonstrating a PVC (indicated by an arrow) **(C)**. A Homo mouse ECG exhibiting a premature atrial contraction (PAC) (indicated by an arrow) and sinus pauses **(D)**. A Homo mouse ECG exhibiting sinus pauses, and sinus bradycardia **(E)**.

**FIGURE 3:**
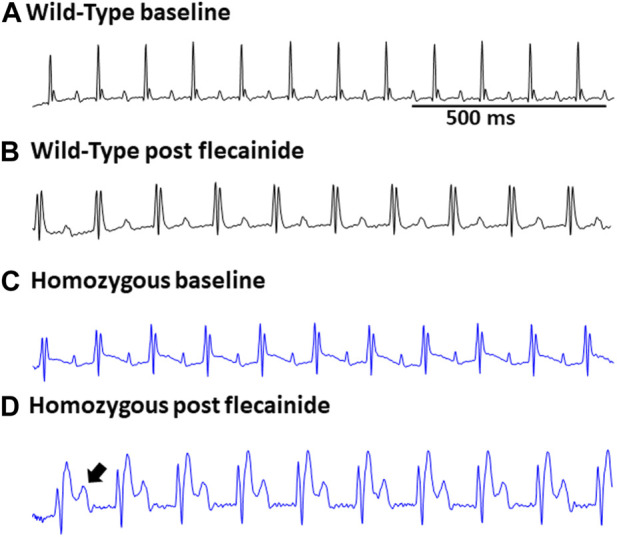
Flecainide heightened susceptibility to ECG abnormalities in DMSXL mice: Representative ECG of wild type at baseline **(A)** and wild type post flecainide **(B)**. Representative ECG of baseline homozygous **(C)** and homozygous after administration of flecainide **(D)**. The flecainide-treated readings show a lengthened PR interval, indicative of a first-degree AV block, and an increased P wave duration. Flecainide was administered intraperitoneally at a dosage of 20 mg/kg body weight.

Surface ECGs were obtained from a total of 17 WT (male n = 9 vs. female n = 8), 16 Het (male n = 8 vs. female n = 8), and 16 Homo (male n = 8 vs. female n = 8) anesthetized mice. WT mice showed a normal sinus rhythm (Figure 2A). 1 out of 16 (6.2%) Het mice ECG showed premature ventricular contractions (PVCs) (Figure 2B) and 3 out of 16 (18.8%) Homo mice showed PVCs, (Figure 2C). Premature atrial contractions (PACs) (Figures 2C,D) were also observed in 2 out of 16 Homo mice. Furthermore, sinus pauses and sinus bradycardia were observed in 2 out of 16 Homo mice (Figures 2D,E). To reveal potentially undetected electrical conduction abnormalities, flecainide, a class-I antiarrhythmic drug, was administered was to both WT (n = 6), Het (n = 4), and Homo mice (n = 5). Representative traces of WT and Homo ECGs at baseline and post-flecainide administration are shown in Figure 3. Administration of flecainide resulted in extended PR, QRS, and QT intervals in both WT and Homo mice (Figures 3A–D). However, the exact values for PR, QRS, and QT could not be determined specifically for the Homo mice as the P wave overlapped with T wave (Figure 3D black arrow). Furthermore, the protracted PR duration, indicating a first-degree AV block, is depicted in Figure 3D. Flecainide administration in Het mice compared to WT showed no significant changes in ECG parameters such as HR (WT 410.0 ± 20.97 bpm, n = 5; Het 451.5 ± 12.62 bpm, n = 4), PR (WT 65.74 ± 3.04 ms, n = 5; Het 64.55 ± 3.4 ms, n = 4), QRS (WT 16.43 ± 1.4 ms, n = 5; Het 20 ± 1.3 ms, *p = 0.0694*, n = 4), and QTc (WT 53.02 ± 2.5 ms, n = 5; Het 49.95 ± 4.1 ms, n = 4).

**FIGURE 4:**
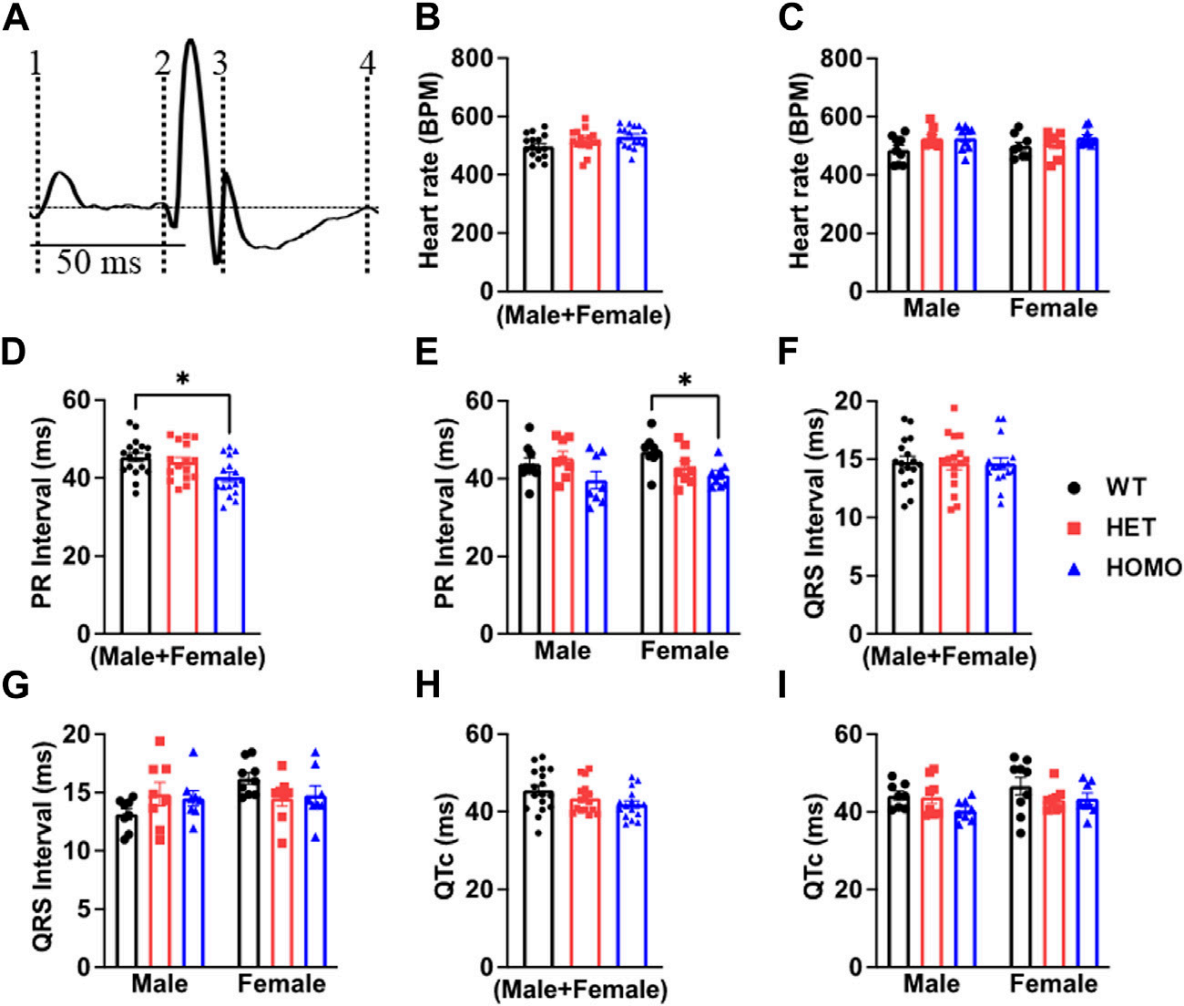
Comparison of ECG parameters between wild type and DMSXL mice: Schematic representation of how ECGs were measured. Dotted line 1 represents the start of the P wave. Dotted line 2 represents the start of the QRS complex. Dotted line 3 represents the end of the QRS complex. Dotted line 4 represents the end of the T wave **(A)**. Both sexes combined heart rate **(B)**, Comparative heart rate distribution among male and female mice of both wild type (WT) and DMSXL (heterozygous (Het) and homozygous (Homo)) **(C)**, Both sexes combined PR interval **(D)**, Comparative PR interval distribution among male and female mice of both WT and DMSXL **(E)**, Both sexes combined QRS interval **(F)**, Comparative QRS interval distribution among male and female mice of both WT and DMSXL **(G)**, Both sexes combined QTc **(H)**, Comparative QTc interval distribution among male and female mice of both WT and DMSXL **(I)**. Bars indicate SEM. *p < 0.05 (WT vs. Het vs. Homo) as determined by ANOVA and Tukey’s *post hoc* test.

Analysis of ECG parameters was performed on tracing sections that did not exhibit arrhythmic events. ECG parameters were defined as indicated in Figure 4A and analyzed as previously described (Boukens et al., 2014). The onset of the P wave was denoted by the initial positive deflection from the isoelectric line (Figure 4A line 1). The beginning of the QRS complex was indicated by the initial downward deviation from the isoelectric line (Figure 4A line 2). The end of the QRS complex was determined when the rising deflection intersected the isoelectric line (Figure 4A line 3). The end of the T wave occurred when the deflection reverted back to the isoelectric line (Figure 4A line 4). When compared to WT, Het and Homo mice showed no change in heart rate between groups (WT 497.60 ± 10.2 bpm, n = 17; Het 516.30 ± 9.7 bpm, n = 16; Homo 528.25 ± 9.1 bpm, n = 16) (Figure 4B). In the sex-specific analysis, Homo male mice showed no change in heart rate compared to WT and Het male mice (WT male 486.42 ± 15.6 bpm, n = 9; Het male 527.00 ± 12.1 bpm, n = 8; Homo male 527.2 ± 13.7 bpm, n = 8, Figure 4C). Conversely, the heart rate of DMSXL females did not significantly differ from that of WT females (WT female 498.8 ± 14.2 bpm, n = 8; Het female 505.5 ± 15.1 bpm, n = 8; Homo female 529.3 ± 10.1 bpm, n = 8, Figure 4C). PR interval was significantly shortened by 10.9% in Homo mice when compared to WT in the sex combined analysis (WT 45.32 ± 1.2 ms, n = 17; Het 44.13 ± 1.2 ms, n = 16; Homo 40.28 ± 1.12 ms, n = 16, Figure 4D). No differences were observed in the male PR interval (Figure 4E). Conversely, the PR interval was remarkably shorter in Homo females compared to WT females (WT female 46.95 ± 1.2 ms, n = 8; Het female 42.97 ± 1.7 ms, n = 8; Homo female 40.94 ± 1.0 ms, n = 8) (Figure 4E). There were no notable variations in the QRS duration between WT and DMSXL mice when analyzing both sexes together (WT 14.77 ± 0.5 ms, n = 17; Het 14.69 ± 0.6 ms; n = 16; Homo 14.64 ± 0.5 ms n = 16 Figure 4F). Furthermore, no significant distinctions were observed in the analysis of sex-specific QRS intervals (Figure 4G). In the sex combined analysis, the QTc in DMSXL mice did not show any significant changes compared to WT mice (WT 45.39 ± 1.1 ms, n = 17; Het 43.55 ± 0.9 ms, n = 16; Homo 41.92 ± 0.9 ms, n = 16) (Figure 4H; Table 1), nor in the sex-specific analysis (Figure 4I; Table 2). To address any potential age differences in ECG parameters in the Homo mice, we performed ECG analyses in 3-month periods (12–14-months, 15–17 months, and 18–20 months). The data shown in the Supplementary Table S1 did not show any statistical differences in electrocardiographic outcomes.

### Ventricular myocytes from DMSXL homo mice exhibited decreased transient outward potassium current densities

**FIGURE 5:**
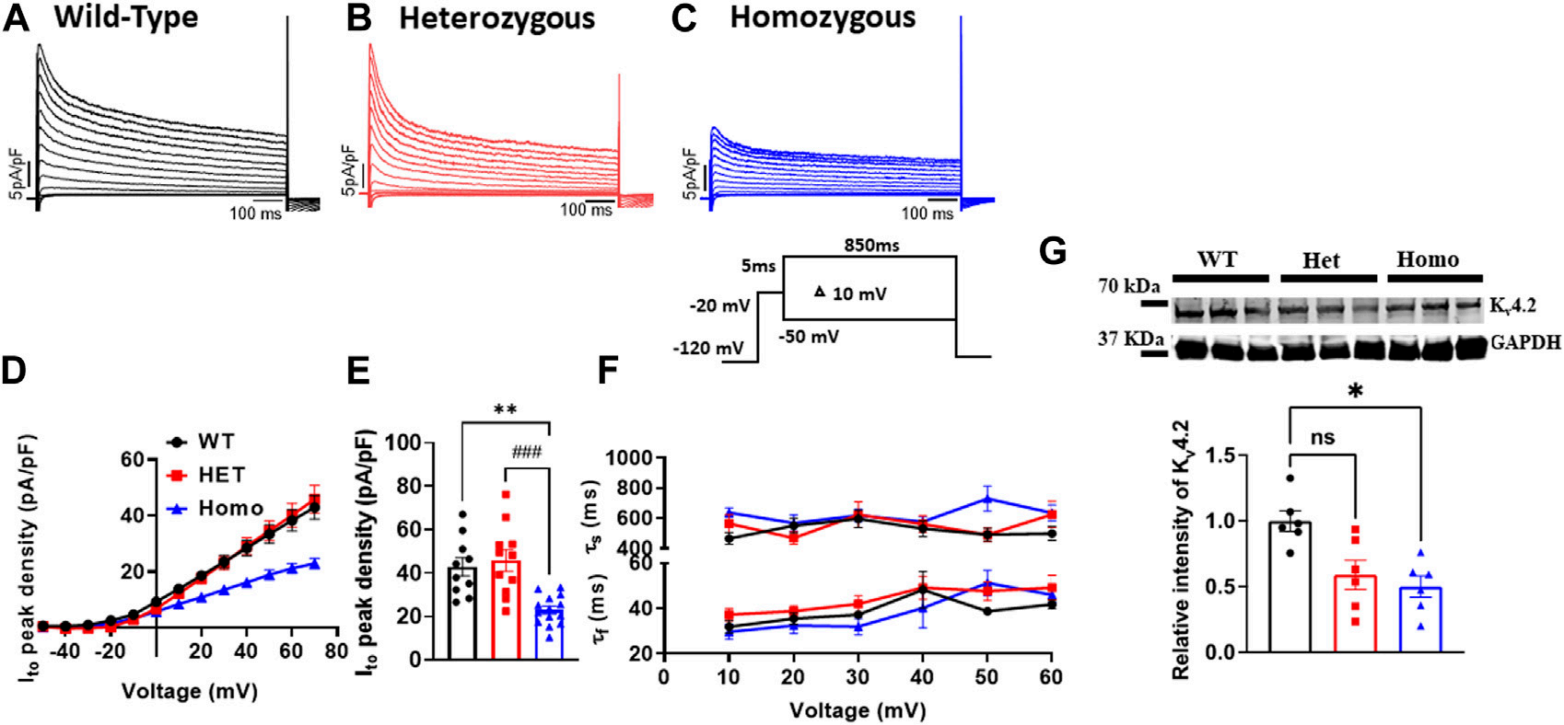
The transient outward K^+^ current (I_to_) density was reduced in ventricular myocytes from DMSXL homozygous mice: Representative I_to_ traces from ventricular myocytes of wild type (WT) **(A)**, heterozygous (Het) **(B)** and homozygous (Homo) **(C)** mice. I_to_ was elicited by the protocol shown in the inset. The current was normalized to the capacitance (pF) of the cells. Current/voltage relationships of I_to_ recorded in ventricular myocytes from WT (n = 10), heterozygous (n = 11), and homozygous (n = 14) **(D)**. Dot plot showing the I_to_ densities recorded at +60 mV **(E)**. The time constants of fast inactivation decay of I_to_ were plotted as a function of voltage for the WT, Het, and Homo ventricular myocytes **(F)**. The time constants were obtained using a double exponential function. A two-exponential function was used to fit the inactivation curves observed on whole cell current traces: I = Afast (exp [- (t - k)/τfast]) + Aslow (exp [- (t - k)/τslow] + **(C)**, where Afast and Aslow are fractions of recovery of the fast and slow components, *t* is time, and *k* is the delay factor for activation or inactivation. Western blot analysis of K_v_4.2 expression. Graph showing K_v_4.2 expression normalized to GAPDH for WT (n = 6), Het (n = 6), and Homo (n = 6) **(G)**. Bars indicate SEM. **p < 0.01; (WT vs. Homo) and ###*p* < 0.001 (Het vs. Homo) as determined by ANOVA and Tukey’s *post hoc* test.

I_to_ plays a prominent role in the initial phase of AP repolarization especially in mice (Xu et al., 1999). Figures 5A–C show representative I_to_ traces recorded from ventricular myocytes of WT, Het, and Homo mice. Peak I_to_ densities were calculated at 70 mV. I_to_ densities from Homo ventricular myocytes were reduced by 46% when compared to WT (WT 42.91 ± 4.2 pA/pF, n = 10; Het 45.87 ± 4.89 pA/pF, n = 11; Homo 22.9 ± 1.8 pA/pF, n = 14 Figures 5D,E and Table 3). There were no significant differences in inactivation kinetics calculated by exponential fitting of the current decays between WT, Het, and Homo (Figure 5F; Table 3). We further investigated the protein expression of potassium voltage-gated channel subfamily D member 2 (K_v_4.2), which contributes to major I_to_ fast in the ventricular tissue. In alignment with the reduction of I_to_ density, we found a decrease in the protein expression level of K_v_4.2 in Homo compared to WT (Figure 5G).

**TABLE 3:** Outward transient potassium (I_to_), L-type calcium (I_CaL_), and sodium current (I_Na_) parameters from wild type and DMSXL ventricular myocytes.

Ionic currents	Wild type	Heterozygous	Homozygous	*p*-value
I_to_ (pA/pF)	42.91 ± 4.23 (n = 10)	45.87 ± 4.89 (n = 11)	22.92 ± 1.81 (n = 14) ** ###	0.0012 (WT vs Homo)
I_CaL_ (pA/pF)	−5.33 ± 0.4 (n = 30)	−4.4 ± 0.6 (n = 12)	−3.76 ± 0.3 (n = 28) **	0.0048 (WT vs Homo)
I_Na_ (pA/pF)	−24.28 ± 2.4 (n = 16)	−19.82 ± 1.7 (n = 9)	−24.09 ± 1.6 (n = 12)	0.9390 (WT vs Homo)

Values are means ± SE. **p < 0.01 (WT vs. Homo) and ###*p* < 0.001 (Het vs. Homo) as determined by ANOVA, and Tukey’s *post hoc* test.

### Ventricular myocytes from DMSXL homo mice exhibited decreased L-type calcium current densities and abnormal gating properties

**FIGURE 6:**
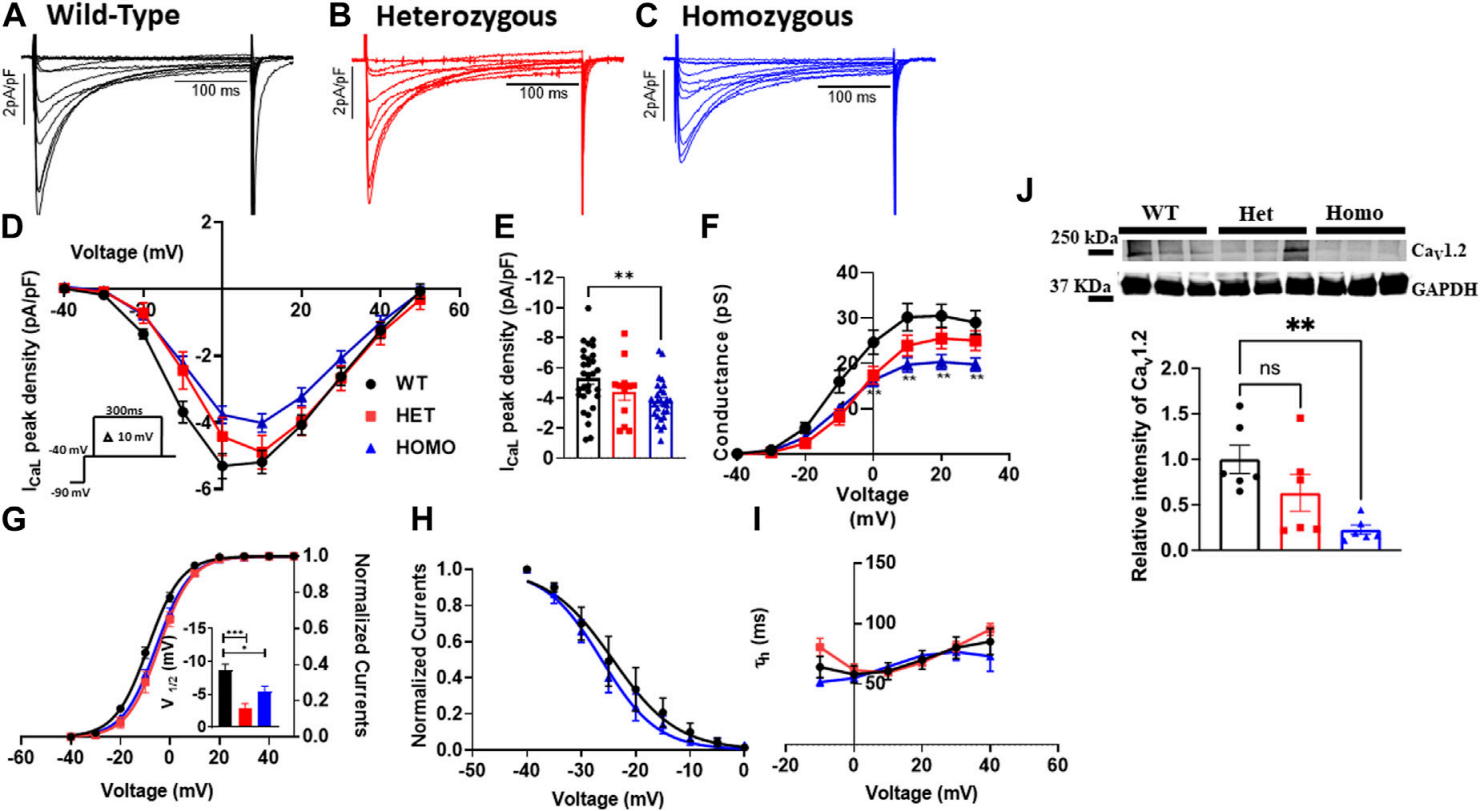
L-type calcium current, (I_CaL_) densities were lower in ventricular myocytes from the DMSXL homozygous mice: Representative I_CaL_ traces recorded from ventricular myocytes of wild type (WT) **(A)**, heterozygous (Het) **(B)** and homozygous (Homo) **(C)** mice. I_CaL_ current/voltage relationships recorded in WT (n = 30), Het (n = 12), and Homo (n = 28) ventricular myocytes **(D)**. The currents were normalized to the capacitance (pF) of the cells. Dot plot showing the I_CaL_ densities recorded at 0 mV **(E)**. Voltage-dependence of steady-state activation were generated by converting I/V into conductance (G/V) **(F)**. Activation curves were then generated using a standard Boltzmann distribution: G(V)/Gmax = 1/(1 + exp (- (V-V_1/2_)/k)). Inset shows graph of V_1/2_ **(G)**. Steady state inactivation of I_CaL_ from WT and Homo **(H)**. The inactivation values were fitted to a standard Boltzmann equation: I(V)/Imax = 1/(1 + exp ((V - V1/2)/k)) + C. The time constants of fast inactivation decay were plotted as a function of voltage for the WT, Het, and Homo ventricular myocytes **(I)**. Western blot analysis of Ca_V_1.2 channel expression. Graph showing Ca_V_1.2 channel expression normalized to GAPDH for WT (n = 6), Het (n = 6), and Homo (n = 6) **(J)**. The time constants were obtained using a single exponential function (A (exp (-t/τ) + C). Bars indicate SEM. *p < 0.05; **p < 0.01; ***p < 0.001; ****p < 0.0001 (WT vs. Het vs. Homo) as determined by ANOVA and Tukey’s *post hoc* test.

Excitation-contraction coupling in cardiac muscle is mediated by L-type calcium channels, which conduct the I_CaL_ that initiates the release of Ca^2+^ from the sarcoplasmic reticulum leading to cardiac contraction. Thus, we measured I_CaL_ in ventricular myocytes of WT and DMSXL mice (Het and Homo) (Figures 6A–C, respectively). Interestingly, a 29% reduction in I_CaL_ density at 0 mV was observed in the I_CaL_ recorded from ventricular myocytes of Homo mice compared to WT, but no significant differences were observed in Het (WT -5.3 ± 0.4 pA/pF, n = 30; Het −4.53 ± 0.53 pA/pF, n = 12; Homo −3.76 ± 0.3 pA/pF, n = 28, Figures 6D,E and Table 3). The I_Ca_ current/voltage (I/V) relationship for the Het and Homo ventricular myocytes revealed a shift toward more depolarized voltages. Ca^+^ conductance was significantly reduced in ventricular myocytes of Homo mice compared to WT (Figure 6F). I_CaL_ activation revealed that the V_1/2_ of the voltage-dependence of I_CaL_ activation of Homo and Het were significantly more positive (WT V_1/2_ = – 8.7 ± 0.9 mV; Het V_1/2_ = – 2.9 ± 0.63 mV; Homo V_1/2_ = – 5.4 ± 0.8 mV, inset shows the graph of V_1/2_ Figure 6G), while the slopes of the curves were similar for all conditions (WT kv = 6.14 ± 0.2; Het kv = 6.02 ± 0.4; Homo kv = 6.4 ± 0.5). However, the inactivation of these Ca^2+^ channels was the same for the Homo and WT ventricular myocytes (Figure 6H). There were no significant differences in inactivation kinetics at hyperpolarized voltages of WT, Het, and Homo, which were calculated by fitting the current decays with an exponential function (Figure 6I). The reduction in I_CaL_ density was confirmed by Western blot, where a significant decrease in L-type calcium channel (Ca_v_1.2) protein expression level was observed in ventricular tissue of Homo mice compared to WT (Figure 6J).

### No change in sodium channel properties in DMSXL mouse ventricular myocytes

**FIGURE 7:**
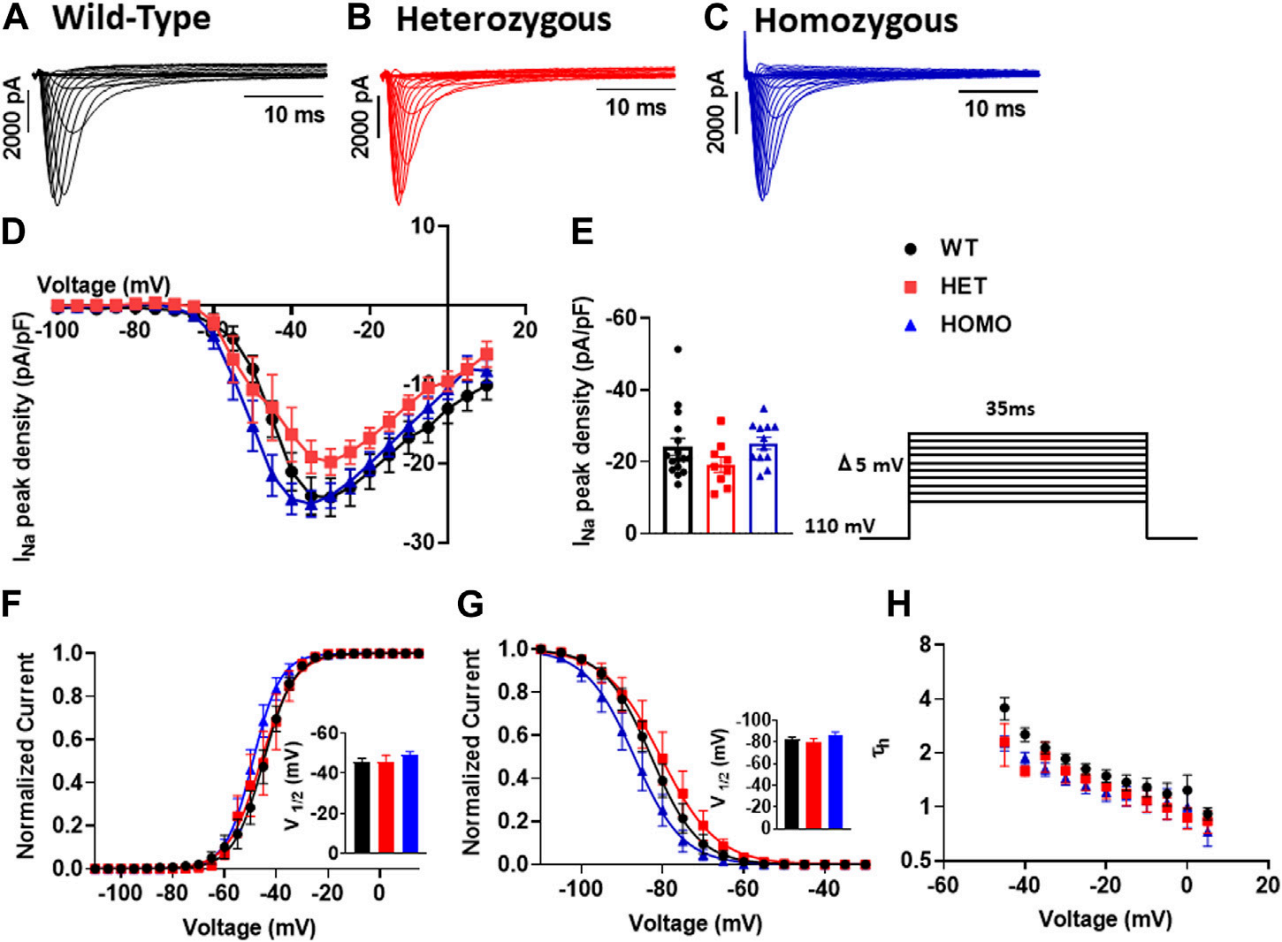
Sodium channels exhibited no electrophysiological changes in DMSXL mice ventricular myocytes: Representative I_Na_ traces recorded from ventricular myocytes of wild type (WT) **(A)**, heterozygous (Het) **(B)** and homozygous (Homo) **(C)** mice. Normalized current/voltage relationships recorded in WT (n = 16), Het (n = 9), and Homo (n = 12) ventricular myocytes **(D)**. I_Na_ densities were measured by normalizing current amplitudes (pA) to cell membrane capacitance (pF) and were plotted against voltage. Dot plot showing the I_Na_ densities recorded at +35 mV **(E)**. Steady-state activation of I_Na_ from WT, Het, and Homo ventricular myocytes **(F)**. Activation curves were generated using a standard Boltzmann distribution: G(V)/Gmax = 1/(1 + exp (- (V-V_1/2_)/k)). Inset shows graph of V_1/2_. Steady state inactivation of WT, Het, and Homo ventricular myocytes **(G)**. The time constants of fast inactivation decay were plotted as a function of voltage for the WT, Het, and Homo ventricular myocytes **(H)**. The time constants were obtained using a single exponential function: (A (exp (- t/τ) + C). Bars indicate SEM. *p*-values (Homo vs. Het vs. WT) as determined by ANOVA and Tukey’s *post hoc* test.

In cardiomyocytes, the I_Na_ initiates and generates the rise of APs. To determine whether I_Na_ is affected in DMSXL ventricular myocytes, we performed patch-clamp analysis in isolated, single ventricular myocytes (Figures 7A–C). We found no significant differences in I_Na_ densities in ventricular myocytes between WT, Het, and Homo groups (WT -24.28 ± 2.4 pA/pF, n = 15; Het −19.82 ± 1.7 pA/pF, n = 9; Homo −24.09 ± 1.6 pA/pF, n = 12; Figures 7D,E and Table 3). To further study the I_Na_ activation, the I/V curves were converted to conductance and were plotted against voltage (G/V). The curves were then fitted using a Boltzmann function. We found no change in the activation between the WT, Het, and Homo ventricular myocytes (WT V_1/2_ = −45.53 ± 1.9 mV; Het V_1/2_ = – 45.9 ± 3 mV; Homo V_1/2_ = – 48.98 ± 1.8 mV; Figure 7F). The steady-state inactivation was then analyzed. No significant shifts were observed between the WT, Het, and Homo ventricular myocytes (WT V_1/2_ = - 82.7 ± 1.8 mV; Het V_1/2_ = - 80.3 ± 2.9 mV, Homo V_1/2_ = - 86.99 ± 2.2 mV; Figure 7G). There were no significant changes in inactivation kinetics at hyperpolarized voltages of WT, Het, and Homo which were calculated by fitting the current decays with exponential functions (Figure 7H).

### Ventricular myocytes from DMSXL homo exhibited no change in action potential duration

**FIGURE 8:**
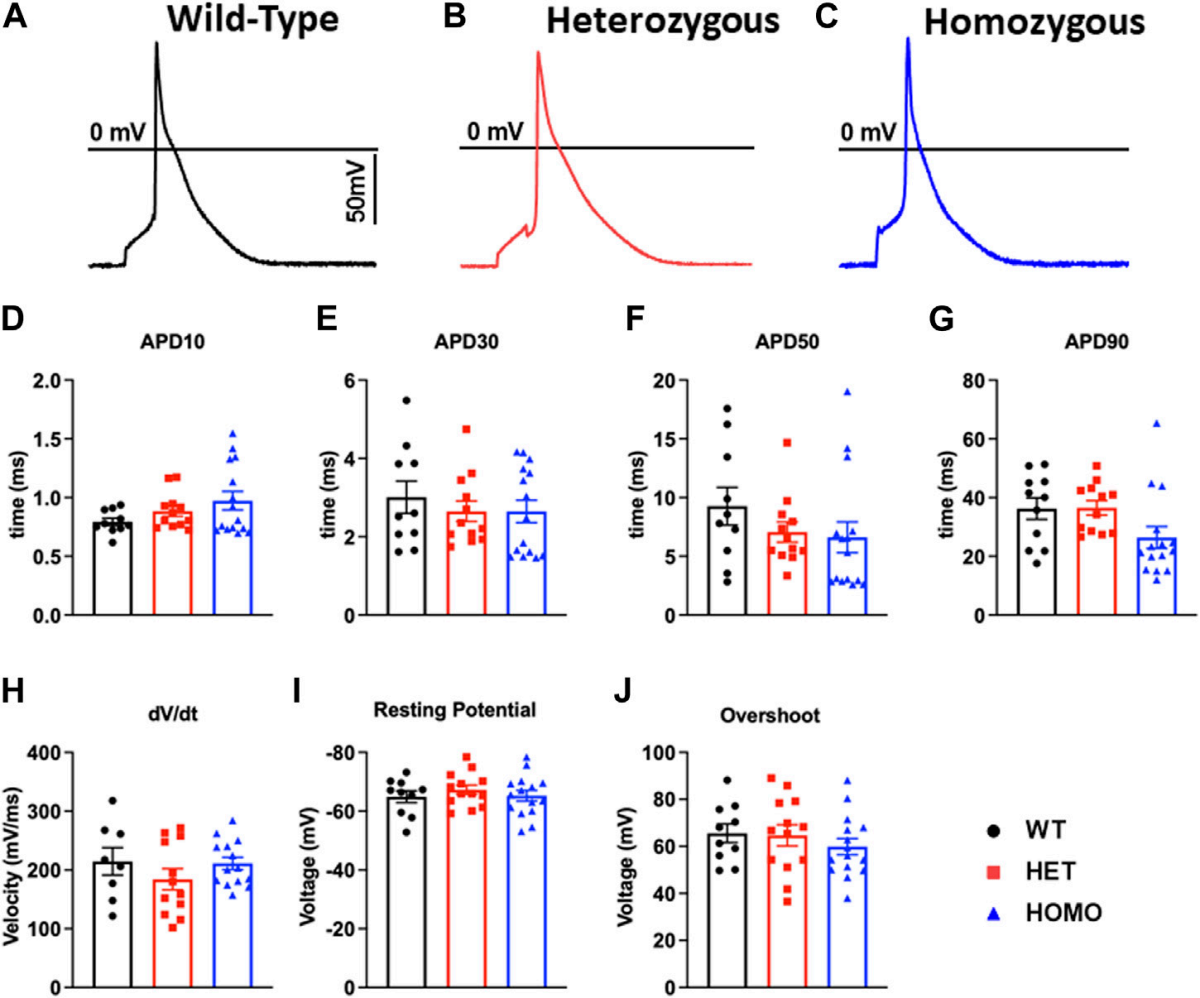
Action potential duration showed no changes between DMSXL and WT ventricular myocytes: Representative action potential (AP) traces of ventricular myocytes from wild type (WT) **(A)**, heterozygous (Het) **(B)** and homozygous (Homo) **(C)** ventricular myocytes. Bar graph summarizing the action potential duration (APD) at 10% **(D)**, bar graph summarizing the APD at 30% **(E)**, bar graph summarizing the APD at 50% **(F)**, bar graph summarizing the APD at 90% **(G)** repolarization in ventricular myocytes (n = 11–15). Bar graphs showing the maximal upstroke velocity (dV/dt_max_) **(H)**, Overshoot **(I)**, and resting membrane potential **(J)**. Bars indicate SEM.

Recordings of APs were obtained from individual isolated ventricular myocytes in WT, Het, and Homo mice to gain an understanding of the mechanism responsible for DM1 cardiac arrhythmogenesis. Figures 8A–C show representative APs recorded from ventricular myocytes isolated from WT, Het, and Homo, respectively. Figure 8 shows bar graphs comparing the action potential duration (APD) measured in WT (n = 10), Het (n = 12), and Homo (n = 15) cells at 10% (APD_10_) Figure 8D, 30% (APD_30_) Figure 8E, 50% (APD_50_) Figure 8F, and 90% (APD_90_) Figure 8G of the repolarization. There were no significant changes in APD_30_ (WT, 3.0 ± 0.4 ms; Het 2.7 ± 0.3 ms; Homo 2.6 ± 0.3 ms), APD_50_ (WT 9.3 ± 1.6 ms; Het 7.1 ± 0.9 ms; Homo 6.6 ± 1.3 ms), and APD_90_ (WT 36.27 ± 3.7 ms; Het 36.53 ± 2.4 ms; Homo 26.44 ± 3.7 ms; Table 4). APs recorded in ventricular myocytes of Homo mice revealed no change in their upstroke velocity (dV/dt) (Figure 8H), overshoot (Figure 8I), and resting membrane potential (Figure 8J) compared to Het and WT.

**TABLE 4:** Action potential duration (APD) from wild type and DMSXL.

	Wild type (n = 11)	Heterozygous (n = 12)	Homozygous (n = 15)
APD_90_ (ms)	36.27 ± 3.7	36.53 ± 2.4	26.44 ± 3.7
APD_50_ (ms)	9.3 ± 1.6	7.1 ± 0.9	6.6 ± 1.3
APD_30_ (ms)	3.0 ± 0.4	2.7 ± 0.3	2.6 ± 0.3
APD_10_ (ms)	0.79 ± 0.03	0.88 ± 0.04	0.97 ± 0.07
Resting Potential (mV)	−64.93 ± 1.9	−67.27 ± 1.6	−65.27 ± 1.8
Overshoot (mV)	65.60 ± 4.0	64.71 ± 4.5	59.90 ± 3.4

Values are means ± SE. *p*-values (WT, vs. Het vs. Homo) as determined by ANOVA, and Tukey’s *post hoc* test.

## Discussion

We conducted a comprehensive electrophysiological assessment, examining both DMSXL and WT mice *in vivo* and DMSXL and WT ventricular myocytes *in vitro*. At the *in vivo* level, the data from the present study showed a growth retardation and ECG abnormalities in Homo mice, and ion channel alterations were found in single ventricular myocyte level. ECG analysis from Homo mice revealed arrhythmic events such as PVCs, PACs, sinus pauses, and sinus bradycardia. In addition, during acute sodium channel blockade with flecainide, an agent used for revealing sodium channel changes in pathological models, Homo mice exhibited an increased susceptibility to arrhythmias. Homo mice also exhibited a significant decreased PR interval and no change in QTc and QRS interval when compared to WT. At the cellular level, Homo mice showed lower densities of I_to_ and I_CaL_ in ventricular myocytes compared to WT. There were no significant differences in I_Na_ densities. These observations offer new perspectives on the arrhythmogenesis and potential therapeutic targets for DM1-related cardiac arrhythmia and could aid in the formulation of novel treatment approaches for patients with DM1.

### Growth retardation in DMSXL homo

Homo mice showed reduced body weight when compared to WT and Het which is consistent with the dystrophic phenotype and with previous work (Algalarrondo et al., 2015). Although growth delay can be of multifactorial origins in humans, low body weight has been reported previously in DM1 patients (Kikuchi et al., 2022). The DMSXL mouse model displays analogous phenotypes to those observed in DM1 patients (Seznec et al., 2000; Huguet et al., 2012; Panaite et al., 2013), rendering it a favorable model for exploring novel therapeutic targets and conducting comprehensive DM1 research. Furthermore, these mice can sustain DM1 phenotypes for more than 20 months, making them suitable for conducting long-term investigations.

### Electrocardiographic abnormalities of DMSXL transgenic mice

The ECG recordings of Homo mice aged between 12 and 20 months revealed electrocardiographic abnormalities like PVCs, PACs, and sinus node dysfunction. These findings, which demonstrate the presence of ECG abnormalities at baseline, are novel and enhance the applicability of this model. Similar arrhythmic incidents observed in our DMSXL mice were also reported in a DM1 mouse model where a non-muscle splice isoform of RNA-binding protein, RBFOX2, was upregulated in the heart (Misra et al., 2020). On the other hand, a previous study conducted on younger DMSXL mouse aged 3 and 8 months did not exhibit any cardiac arrhythmic events at baseline (Algalarrondo et al., 2015). Previous investigations have suggested that age may be a critical factor in the onset of arrhythmias in DM1 patients, as previously documented (Harper et al., 1992; Hunter et al., 1992), which could explain this phenomenon. It is worth noting that not all DM1 patients have abnormal ECGs. For instance, Lazarus et al. observed paroxysmal bradycardia in only 23 out of the 45 patients studied, with only 5 individuals displaying spontaneous complete permanent AV blocks over a mean follow-up duration of 54 ± 27 months (Lazarus et al., 2002). Therefore, it is anticipated that electrical anomalies would be seen in only a subset of both DM1 patients and DMSXL mice. In this study, we also did not observe any age-related changes in ECG parameters in the Homo mice within our cohort (12–20 months) indicating that the 8-month age range in did not impact the ECG parameters.

In our investigations into flecainide sensitivity, we noticed a prolongation in the P duration, PR interval, QRS interval, and QTc interval in both WT and Homo mice, as shown in the ECG analysis. However, the ECG of Homo mice revealed a more noticeable prolongation of the P duration and PR interval and first-degree AV block. The expanded P wave and PR interval intersected with the T wave, thereby hindering accurate analysis and statistical evaluation. Our findings regarding flecainide-induced arrhythmia in the DMSXL mouse model are consistent with previous studies (Algalarrondo et al., 2015; Tylock et al., 2020). The challenge in conducting an analysis of ECG parameters in Homo mice is due to the collision of the P wave with the T wave as a result of a severe prolongation of the P duration and PR interval compared to WT mice. This affirms that this older model exhibits an enhanced sensitivity to flecainide, making it a more suitable model for investigating therapeutic strategies. Baseline ECG in Homo mice also showed a shorter PR interval, but normal QRS duration and QTc, which is consistent with a prior study on these mice (Algalarrondo et al., 2015). However, our results are not consistent with a study in LC15 DM1 mice, which showed increased QTc in LC15 DM1 mice compared to WT mice (Tylock et al., 2020). The PR interval changes were sex-specific and observed only in females. Although sex hormones are known to affect ionic currents as reflected in QT interval studies (Sedlak et al., 2012; Bjelic et al., 2022), no previous work has addressed the sex hormone effects on PR interval, warranting future studies. The exact basis for a shortened PR-interval in our DMSXL mice is unknown, and no literature which could explain this observation is available to date.

### Transient outward potassium current density is reduced in ventricular myocytes from DMSXL mice

As mentioned earlier, I_to_ causes the initial phase of repolarization of the AP in ventricular myocytes (Xu et al., 1999). A significant reduction in the peak density of I_to_ was observed in ventricular myocytes from Homo mice compared to WT and Het (Figure 5). A notable decrease in the expression of the K_v_4.2 channel, one of the major contributors to I_to_ fast, within the ventricular tissue of Homo mice might explain the reduced I_to_ density at the single ventricular myocyte level in Homo mice. These findings imply abnormal I_to_ may be a potential therapeutic target. The reduction in I_to_ density may be linked to slight but not statistically different increase in APD_10_ of the AP (Figure 8A) as one might expect. However, the lack of significance could be due to the AP shortening (Figure 8D) caused by the reduced I_CaL_ density (Figure 6) counterbalancing the lengthening of AP anticipated by the reduction in I_to_ densities. In a recent study, RBFOX2 overexpression in mice, mimicking the RBFOX2 overexpression seen in human DM1 patients, triggered splicing defects in sodium and potassium channels (K_v_4.3), which could potentially explain the reduced I_to_ seen in Homo mice (Misra et al., 2020). A different study on iPSC-derived cardiomyocytes (iPSC-CM) from a DM1 patient showed that the *KCND3* gene, which encodes K_v_4.3 and is responsible for I_to_, had lower expression levels compared WT (Spitalieri et al., 2018). Another recent study observed I_to_ decrease in ventricular myocytes from the LC15 DM1 mouse models having expanded CUG RNA repeats (Tylock et al., 2020). Their results support our findings, indicating a reduction in the density of I_to_ in LC15 mouse model (Tylock et al., 2020). The decreased I_to_ appears to be more accentuated in ventricular myocytes of DMSXL mice compared to LC15 mice, with a reduction of 47% in DMSXL mice (Homo 22.9 ± 1.8 pA/pF vs. WT 42.91 ± 4.2 pA/pF) and 22% in LC15 mice (LC15 32.5 ± 1.9 pA/pF vs. WT 41.6 ± 2.6 pA/pF). These findings reinforce the interest in I_to_ as a potential therapeutic target. Noteworthy is the major difference between the DMSXL and LC15 models in that the LC15 mice contain a smaller (250–400) CTG insert and lack other key DM1 phenotypes such as decreased motor performance and primary DM1 mechanisms such as MBNL1 sequestration (Seznec et al., 2000; Algalarrondo et al., 2015; Tylock et al., 2020). Therefore, DMSXL positions itself to be a more appropriate model to study DM1 as it better recapitulates the clinical phenotype.

### L-type calcium current density was reduced and abnormal gating properties were found in DMSXL homo mouse ventricular myocytes

The impact of L-type calcium channels on cardiac electrical abnormalities in the DMSXL model has not been previously investigated. Our study provides evidence, *for the first time*, of substantial decreases in I_CaL_ density in ventricular myocytes isolated from Homo mice. Specifically, the I_CaL_ was reduced by 28% when compared to WT, a novel observation in the DMSXL mouse model of DM1 (Figures 6D,E). We also observed a significant shift in the activation toward depolarized potentials (Figure 6F). Downregulation of the Ca_v_1.2 channel protein expression within the ventricular tissue of Homo mice could potentially account for the reduced I_CaL_ in these mice. Our results are supported by a recent study in hiPSC-CMs derived from DM1 patients, which showed a significant decrease in the expression of the gene encoding L-type calcium channel Ca_v_1.2, *CACNA1C* (Spitalieri et al., 2018). In our recent study (unpublished data) we showed a decrease in I_CaL_ in iPSC-CMs from DM1 patients. However, in our previous study using different DM1 derived iPSC-cardiomyocytes we found increased I_CaL_ (Poulin et al., 2021) indicating patient-specific differences in generating iPSC-cardiomyocytes. No other studies on DM1 mouse models reported on the role of I_CaL_ in the pathogenesis of DM1. The observed positive shifts in activation and V_1/2_ indicate a change in the voltage dependence of the L-type calcium channels. Gating defects could arise from alternative splicing or modifications that affect the conformational changes necessary for channel activation and conductance. Such changes can hinder channel opening or favor channel closure, leading to reduced I_CaL_.

### No change in sodium channel properties in DMSXL mouse ventricular myocytes

The proper functioning of cardiac APs heavily relies on cardiac sodium channels, which is essential for both the initiation and propagation (Coraboeuf et al., 1979; Pierre et al., 2021). The cardiac sodium channel’s biophysical characteristics significantly influence the phase 0 of APs and regulate APDs. We did not observe any changes in I_Na_ densities, activation, and inactivation properties in ventricular myocytes of older Homo mice (12–20months, Figure 7). In the Algalarrondo et al. study, I_Na_ density, activation, and channel availability were unchanged in ventricular myocytes of DMSXL mice (3 and 8 months), but the recovery from inactivation was quicker in the ventricular myocytes from DMSXL mice compared to WT mice (Algalarrondo et al., 2015). These differences could be due to mice age.

### DMSXL homo exhibited no change in action potential duration

The occurrence of arrhythmic events in the ECG of Homo mice and the reduction in I_to_ and I_CaL_ densities in the ventricular myocytes led us to anticipate significant modifications in AP properties. However, our findings from AP recordings in isolated ventricular myocytes of Homo mice were surprising, as there was no alteration in the APD (Figure 8), consistent with the results reported by Algalarrondo et al. (Algalarrondo et al., 2015). Similarly, Poulin et al. showed no differences in APD recorded in DM1 patient derived iPSC-ventricular myocytes (Poulin et al., 2021). However, a study (Tylock et al., 2020) using the LC15 DM1 mouse model showed significant increases in APD and QTc as one would expect. Although the LC15 study showed a significant decrease in I_to_ in ventricular myocytes, which could account in part for the APD prolongation, no data on I_CaL_ was reported making it difficult to assess the basis for the APD prolongation. As such, a direct comparison with our study findings, which demonstrated a decrease in both I_to_ and I_CaL_ resulting in net zero effect on APD and QTc, is challenging. To this end, studies have demonstrated that a decrease in outward potassium channels can lead to an increase in APD (Mitterdorfer and Bean, 2002). Conversely, a loss of function in L-type calcium channels can cause a shortened APD (Ficker et al., 2004). When the cardiac L-type calcium channels and outward potassium channels experience loss of function, they may offset each other’s effect on the APD. This phenomenon could potentially explain why previous research on DM1 has not consistently shown prolonged APD in ventricular myocytes derived from both mice and iPSCs models.

### Potential mechanisms for arrhythmic events on DMSL mice

The main electrical abnormalities observed in the DMSXL mice are PVCs and PACs with the potential to initiate ventricular arrhythmias. Although the pathophysiology of PVCs and PACs is still not well established, the most cited causes are triggered activity, automaticity, re-entrant arrhythmias and ion channel dysfunction (Guichard et al., 2022; Marcus, 2022). While triggered activity is associated with afterdepolarizations due to intracellular calcium dysfunction, which may initiate arrhythmia, automaticity can be ectopic with the potential for firing an AP leading to arrhythmic events. PVCs due to re-entrant arrhythmias can originate from the Purkinje fibers in the presence of a slower conduction and/or a block leading to the firing of a beat on the post block pathway, and PACs can originate from the pulmonary veins. Structural remodeling, including fibrosis and autonomic nervous system abnormalities, can also play an important role in the occurrence of PACs and PVCs. In this study, while we found dysfunctional ion channels such as I_CaL_ and I_to_ the exact mechanism (s) responsible for the observed arrhythmic events in mice and humans warrant further investigations.

### Study limitation

Although the present study provided new insights into the electrocardiographic abnormalities in the DMSXL mice model of DM1 which exhibited spontaneous PACs, PVCs, and sinus pause/bradycardia, these mice did not show conduction abnormalities such as different degrees of AV block seen in DM1 patients. These discordances could be due to the electrophysiological differences between mice and humans, such as the 10-fold faster heart rate, significantly shorter APD, and difference in ion channel expression in mice compared to humans. Furthermore, the accurate assessment of the prevalence of arrhythmic events in mice would require weeks of continuous ECG monitoring which was not performed in this study. Sinus bradycardia was observed in only a few Homo mice (2 out of 16) and the overall heart rate values did not significantly change between the Homo and WT mice indicating that the pacemaker current, I_f_ is unlikely to play a major role in the sinus node dysfunction. Collectively, the reduction in I_to_ and I_CaL_ densities and protein levels at the cardiac myocyte level provide important insights, however, the exact mechanisms of increased arrhythmic events in DMSXL mice remain to be elucidated.

## Conclusion

In conclusion, our investigation employing the DMSXL DM1 mouse model unveiled substantial dysregulation in cardiac ion channels. These dysfunctions include the loss of the transient outward potassium and L-type calcium channel function, as well as abnormal gating properties of L-type calcium. These ion channel abnormalities may contribute to arrhythmic events observed in DMSXL mice and may have implications for the clinical phenotype of DM1 patients. Our electrophysiological findings offer crucial insights into therapeutic targets that could be developed using the DMSXL mouse model, with a specific focus on L-type calcium, the transient outward potassium, and their gating properties.

## Data Availability

The raw data supporting the conclusion of this article will be made available by the authors, without undue reservation.

## Abbreviations

AP, Action Potential; APD, Action Potential Duration; AV, Atrioventricular; *CACNA1C*, Voltage-gated calcium channel gene; CTG, Cytosine-thymine-guanine; DM1, Myotonic dystrophy type 1; DMPK, Dystrophia Myotonia Protein Kinase; ECG, electrocardiogram; Het, heterozygous; Homo, homozygous; I_CaL_, L-type calcium current; I_CaT_, T-type calcium current; I_Na_, Sodium current; iPSC, Induced pluripotent stem cells; I_to_, Transient outward potassium current; I/V, Current–voltage relationship; PVC, Premature ventricular contractions; PAC, Premature atrial contractions; QTc, Heart rate corrected QT interval; SCD, Sudden cardiac death; UTR, Untranslated region; WT, Wild type.
