# Supplementary material for: Electrophysiological basis of cardiac arrhythmia in a mouse model of myotonic dystrophy type 1
Source: Front Physiol. 2023 Sep 21;14:1257682. doi: 10.3389/fphys.2023.1257682 (PMC10551179; doi:10.3389/fphys.2023.1257682)
Supplement: Supplementary file 1 [file Table1.DOCX]

**Supplementary Table 1: Genotyping of DMSXL mice**

| Mouse ID | Intensity | Zygosity | Sex |
| --- | --- | --- | --- |
| 34L | 2.5572 | Homozygous | Female |
| 38N | 2.21265 | Homozygous | Female |
| 50N | 2.4092 | Homozygous | Female |
| 61L | 2.2817 | Homozygous | Female |
| 64L | 2.9436 | Homozygous | Female |
| 64N | 3.0525 | Homozygous | Female |
| 71L | 1.976 | Homozygous | Female |
| 77N | 2.84075 | Homozygous | Female |
| 79B | 2.51555 | Homozygous | Female |
| 83L | 1.9848 | Homozygous | Female |
| 72N | 1.962 | Homozygous | Female |
| 29R | 2.22 | Homozygous | Male |
| 32N | 2.9508 | Homozygous | Male |
| 37B | 2.45195 | Homozygous | Male |
| 40N | 3.8575 | Homozygous | Male |
| 40R | 2.51555 | Homozygous | Male |
| 56L | 2.08255 | Homozygous | Male |
| 57N | 2.08255 | Homozygous | Male |
| 58R | 2.84075 | Homozygous | Male |
| 63L | 2.08255 | Homozygous | Male |
| 63N | 3.01005 | Homozygous | Male |
| 82B | 1.90075 | Homozygous | Male |
| 84R | 3.0589 | Homozygous | Male |
| 87N | 1.9179 | Homozygous | Male |
| 30R | 1.2039 | Heterozygous | Female |
| 34N | 1.4527 | Heterozygous | Female |
| 36N | 1.38615 | Heterozygous | Female |
| 61N | 1.35785 | Heterozygous | Female |
| 61R | 1.231 | Heterozygous | Female |
| 65N | 1.7249 | Heterozygous | Female |
| 65R | 1.43135 | Heterozygous | Female |
| 70B | 1.3863 | Heterozygous | Female |
| 75L | 1.06865 | Heterozygous | Female |
| 75N | 1.2524 | Heterozygous | Female |
| 78B | 1.04615 | Heterozygous | Female |
| 95N | 1.26215 | Heterozygous | Female |
| 95L | 1.2616 | Heterozygous | Female |
| 51N | 1.4513 | Heterozygous | Female |
| 29N | 1.07785 | Heterozygous | Male |
| 40L | 1.07025 | Heterozygous | Male |
| 69L | 1.22895 | Heterozygous | Male |
| 69R | 1.52285 | Heterozygous | Male |
| 71N | 1.48785 | Heterozygous | Male |
| 72L | 1.5368 | Heterozygous | Male |
| 74L | 1.28655 | Heterozygous | Male |
| 74N | 1.41985 | Heterozygous | Male |
| 77B | 1.79805 | Heterozygous | Male |
| 81N | 1.3348 | Heterozygous | Male |
| 84L | 1.75425 | Heterozygous | Male |
| 84N | 1.5641 | Heterozygous | Male |
| 85N | 1.3948 | Heterozygous | Male |
| 85R | 1.5078 | Heterozygous | Male |
| 87R | 1.37325 | Heterozygous | Male |
| 91N | 1.25215 | Heterozygous | Male |
| 73L | 0 | Wild Type | Female |
| 83N | 0 | Wild Type | Female |
| 92L | 0 | Wild Type | Female |
| 95R | 0 | Wild Type | Female |
| 70L | 0.00005 | Wild Type | Female |
| 79L | 0 | Wild Type | Female |
| 93N | 0 | Wild Type | Female |
| 74R | 0 | Wild Type | Male |
| 80N | 0 | Wild Type | Male |
| 86N | 0 | Wild Type | Male |
| 90N | 0 | Wild Type | Male |
| 89B | 0 | Wild Type | Male |
| 89R | 0 | Wild Type | Male |

Wild type intensity range: 0.00-0.01

Heterozygous intensity range: 0.82-1.80

Homozygous intensity range: 1.90-3.86

Additional wild type mice were supplemented with commercially available C57BL/6 mice.

| **Homo** | **12-14 Months (n=6)** | **15-17 Months (n=5)** | **18-20 Months (n=5)** |
| --- | --- | --- | --- |
| **HR** | 536.6 ± 9.36 | 539.3 ± 19.27 | 514.2 ± 20.09 |
| **PR** | 37.74 ± 1.583 | 41.07 ± 1.745 | 42.54 ± 2.795 |
| **QRS** | 14.28 ± 0.1937 | 15.01 ± 1.32 | 14.7 ± 1.093 |
| **QTc** | 41.47 ± 1.533 | 43.1 ± 2.136 | 41.28 ± 1.266 |

**Supplementary Table 2: ECG parameters of homozygous mice at different age breakdowns**

Values means ± SE.
